# Endometriosis Treatment: Role of Natural Polyphenols as Anti-Inflammatory Agents

**DOI:** 10.3390/nu15132967

**Published:** 2023-06-30

**Authors:** Valentina Tassinari, Antonella Smeriglio, Virgilio Stillittano, Domenico Trombetta, Romano Zilli, Roberta Tassinari, Francesca Maranghi, Giulia Frank, Daniele Marcoccia, Laura Di Renzo

**Affiliations:** 1 Department of Experimental Medicine, University of Rome Tor Vergata, Via Montpellier 1, 00133 Rome, Italy; valentina.tassinari@uniroma2.it; 2 Department of Chemical, Biological, Pharmaceutical and Environmental Sciences, University of Messina, Viale Ferdinando Stagno d’Alcontres 31, 98166 Messina, Italy; antonella.smeriglio@unime.it (A.S.); domenico.trombetta@unime.it (D.T.); 3 Istituto Zooprofilattico Sperimentale del Lazio e della Toscana “M. Aleandri”, Via Appia Nuova 1411, 00178 Rome, Italy; v.stillittano_esterno@sanita.it (V.S.); romano.zilli@izslt.it (R.Z.); 4 Center for Gender-Specific Medicine, Istituto Superiore di Sanità, 00161 Rome, Italy; roberta.tassinari@iss.it (R.T.); francesca.maranghi@iss.it (F.M.); 5 Ph.D. School of Applied Medical-Surgical Sciences, University of Tor Vergata, Via Montpellier 1, 00133 Rome, Italy; giulia.frank@ymail.com; 6 School of Specialization in Food Science, University of Rome Tor Vergata, 00133 Rome, Italy; laura.di.renzo@uniroma2.it; 7 Section of Clinical Nutrition and Nutrigenomic, Department of Biomedicine and Prevention, University of Rome Tor Vergata, Via Montpellier 1, 00133 Rome, Italy

**Keywords:** endometrium, anti-inflammatory activity, polyphenols, NSAIDs

## Abstract

Endometriosis is an estrogen-dependent common chronic inflammatory disease defined by the presence of extrauterine endometrial tissue that promotes pelvic pain and fertility impairment. Its etiology is complex and multifactorial, and several not completely understood theories have been proposed to describe its pathogenesis. Indeed, this disease affects women’s quality of life and their reproductive system. Conventional therapies for endometriosis treatment primarily focus on surgical resection, lowering systemic levels of estrogen, and treatment with non-steroidal anti-inflammatory drugs to counteract the inflammatory response. However, although these strategies have shown to be effective, they also show considerable side effects. Therefore, there is a growing interest in the use of herbal medicine for the treatment of endometriosis; however, to date, only very limited literature is present on this topic. Polyphenols display important anti-endometriotic properties; in particular, they are potent phytoestrogens that in parallel modulates estrogen activity and exerts anti-inflammatory activity. The aim of this review is to provide an overview on anti-inflammatory activity of polyphenols in the treatment of endometriosis.

## 1. Introduction

**Figure 1:**
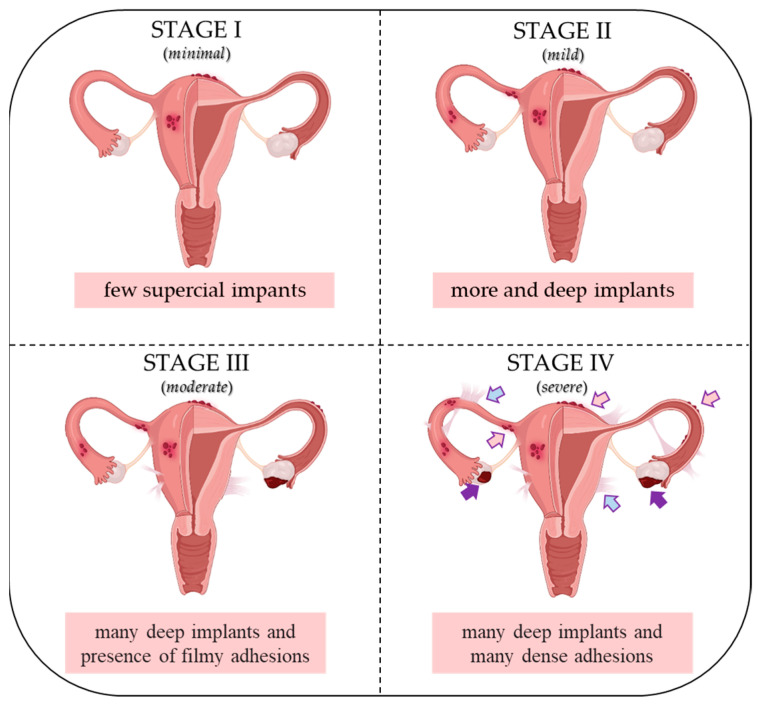
ASRM classification of endometriosis I–IV stages. Pink arrows indicate superficial implants; purple arrows indicate dense adhesions; blue arrows indicate filmy adhesions. Created with biorender.com.

Endometriosis (EMS) is a common estrogen-dependent gynecological disease [1] characterized by the growing of endometrial cells outside the uterus (commonly known as “endometriosis implants”), especially in the pelvic area including the ovaries, ligaments and peritoneal surfaces as well as bowel and bladder [2,3]. Implants promote inflammation, which in more aggressive endometrial stages (III and IV) result in a band of scar tissue, also called endometrial adhesions. EMS affects 10–15% of women in reproductive age and it is associated with different symptoms such as infertility, chronic pelvic pain, dyspareunia, dysmenorrhea and abnormal uterine bleeding [1,2]. The American Society for Reproductive Medicine (ASRM) classifies EMS into four different stages according to the size of the endometriotic lesions in the ovaries, peritoneum, and fallopian tubes, and the severity of adhesion at each of the aforementioned sites: minimal (Stage I), mild (Stage II), moderate (Stage III) and severe (Stage IV) (Figure 1) [4].

EMS diagnosis occurs in 2 to 11% of asymptomatic women, 5 to 50% of infertile women, and 5 to 21% of women hospitalized for pelvic pain. It is interesting to note that EMS occurs also in a small percentage (9%) of adolescents who experience chronic pelvic pain, with the majority of them (75%) not responding to medical treatments [5]. This condition, which heavily impacts the patient’s quality of life, is identified through surgical or laparoscopy exploration, followed by histopathological analyses [6,7].

It is widely known that genetic, immunological, and environmental factors play a pivotal role in the onset of EMS [8,9,10]. Although its etiology is unknown, different studies confirm that it is characterized by changes in the activity of estradiol (E2) and progesterone (P4) receptors, which impairs their dependent pathways and results in P4 resistance and E2 dependence. These events seem to be strongly correlated with pain and infertility in EMS-affected women [10,11]. Indeed, it has been demonstrated that metabolic alterations of sex steroids E2 and P4 affect the ability of endometriotic cells to proliferate, migrate, and infiltrate the mesothelium. Furthermore, they promote the release of pro-inflammatory factors, playing a critical role in disease progression [12,13]. Several studies showed that pro-inflammatory mediators, such as cytokines, metalloproteinases (MMPs) and prostaglandins (PGs), enhance sex steroid receptor down-regulation, increasing aromatase activity (encoded by *CYP19A1* gene), the enzyme mainly responsible for estrogen biosynthesis [14,15,16,17]. Inflammation can activate the eicosanoids pathway through enzymatic and non-enzymatic oxidation of arachidonic acid (AA) produced by phospholipase A2 (PLA2) from the membrane phospholipids. AA is, in turn, metabolized by cyclooxygenases (COXs), lipoxygenases (LOXs) and nitric oxide synthase (NO_S_), giving rise to different oxidation products such as PGs, thromboxanes (TXs), leukotrienes (LTs) and lipoxins (LXs). Cytokines trigger the up-regulation of COX-2 expression; as result, the COX-2-dependent prostaglandin E2 (PGE_2_) biosynthesis is higher in the peritoneal fluid of EMS patients compared to normal endometrium [16,18,19,20,21]. This inflamed micro-environment strongly sustains the proliferation and invasion of the endometrium epithelial and stromal cells, reducing apoptosis and enhancing angiogenesis [22]. As a consequence, the therapeutic inhibition of COX-2, PGE_2_, and/or their receptors—prostaglandin E receptor 2 (PTGER2) and prostaglandin E receptor 4 (PTGER4)—decreases the survival and invasive ability of endometriosis cells [22].

Currently available non-steroidal anti-inflammatory drugs (NSAIDs) can be classified into two main families: (i) non-selective NSAIDs, such as ibuprofen (IBF), naproxen (NAP) and aspirin (ASA) that inhibit COXs-dependent PGs production; and (ii) selective COX-2 inhibitors, such as celecoxib (CXB), mainly used as first-line treatment for EMS-affected women [23,24].

However, these therapeutic agents show short- and long-term adverse effects, especially on the gastrointestinal and cardiovascular systems [24,25,26,27,28], which limit a lot their use in EMS patients.

Considering this, and the always-high incidence of this pathology, there is an increasing interest to explore additional and alternative approaches. From this point of view, several studies have demonstrated the critical role of some non-nutrient compounds such as polyphenols in counteracting EMS-related pro-inflammatory events [29,30] as promising alternative strategies. Furthermore, some natural compounds, such as polyphenols like resveratrol (RESV), might also potentially minimize the adverse effects of NSAIDs [31,32,33,34,35,36], thus potentially exploitable for co-treatment approaches.

This narrative review aims to summarize the current knowledge on the anti-inflammatory properties of natural compounds, focusing on their potential use as NSAID alternatives for the treatment of EMS.

## 2. Materials and Methods

Case reports, original studies and recent reviews published between 2010 and 2023 were collected in scientific databases available online, e.g., PubMed, Web of Science, Google Scholar and Science Direct.

**Figure 2:**
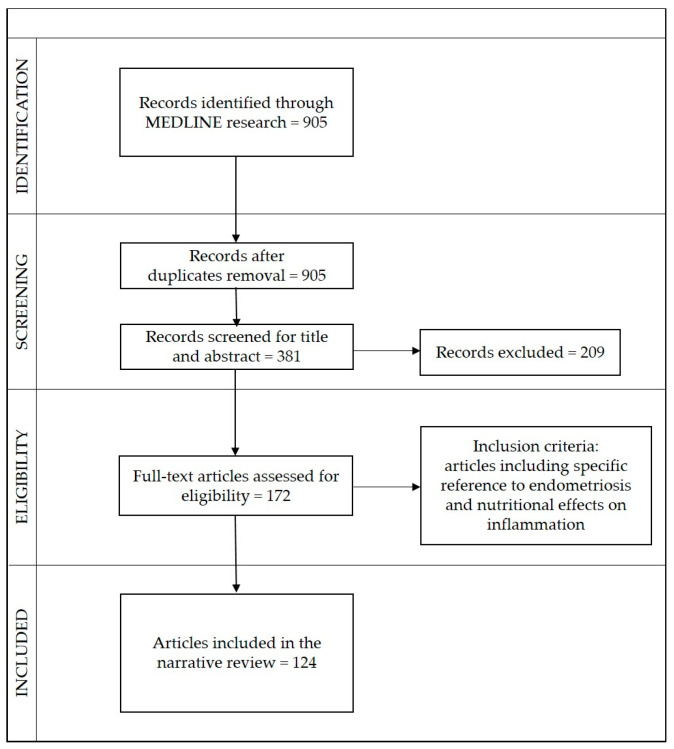
Flow chart meta-analyses (PRISMA).

The study was carried out according to the Preferred Reporting Items for Systematic Reviews and Meta-Analyses (PRISMA) scheme. The selection process, inclusion, and analysis are shown in (Figure 2).

Article titles were double-checked and duplicates were excluded. Overall, 905 articles were identified, of which 524 were excluded after two reviewers’ provisional assessment of titles and abstracts, and 209 after full-text screening. In the end, 124 articles relevant to the topic were selected, including 29 original articles, 81 reviews, and 13 case reports.

## 3. Current and Alternative Treatments of Endometriosis

The therapeutic strategies adopted for EMS patients are strictly dependent on different criteria such as age, side-effect profile, lesion extent and locations, as well as preliminary treatments [37,38]. Nevertheless, the surgery to remove the ectopic endometriosis lesions represents the treatment of first choice, generally followed by long-term pharmaceutical therapy with NSAIDs and oral contraceptives [39,40,41]. Generally, in order to avoid EMS-dependent PGs formation, NSAID-dependent treatment foresees the administration of the drug a few days before the menstruation, thus reducing pain and swelling. NSAIDs inhibit PGE_2_ production through a reversible blockade of COX-1 and COX-2 enzymes, which mainly catalyze the conversion of AA into PGs. Non-selective NSAIDS, like IBF or NAP, block both of these enzymes, while other NSAIDs, such as CXB, only block the COX-2 enzyme.

Oral contraceptives induce hypoestrogenemia in addition to the inhibition of tissue proliferation and inflammation [42]. Progestins plus gonadotropin-releasing hormone agonists (GnRH) that cause amenorrhea is another method for reducing systemic estrogen levels [43], alleviating disease-related symptoms [44]. However, the conventional medical procedures outlined above may result in limited efficacy for the majority of patients due to the onset of several side effects, including perimenopausal stage symptoms, osteoporosis, lipid profile changes, and liver dysfunction [40,41]. Therefore, in order to counteract EMS, many women are using non-pharmacological alternatives mainly based on natural substances that, in addition to a healthy lifestyle characterized by exercise, health nutrition, osteopathy and relaxation techniques like autogenic training and meditation [3,44], allow to properly control and sometimes revert the EMS symptoms.

Polyphenols, which include different classes of flavonoids and stilbenes, are natural substances that have been extensively investigated for their anti-inflammatory properties. They may provide an important alternative to NSAIDs because of their anti-inflammatory activity, the fact that they are often COX-2 selective, and by accelerating the healing process, decreasing the side effects that EMS patients currently experience [3,45].

## 4. Dietary, Nutritional and Molecular Aspects in Endometriosis

Diet is crucial in the development and management of EMS [3,27,46,47] given its ability to control the metabolism of steroid hormones, inflammation, muscle contraction, menstrual cycle and PG metabolism [48]. Interestingly, poor eating habits and deficiency of several nutrients such as folic acid, vitamin B12, zinc and choline may also interfere with the DNA methylation process affecting gene expression, that, in turn, affects the development of EMS [9,48,49]. For example, reduced methylation of the ERβ gene promoter region in endometriotic cells with respect to the healthy endometrial cells results in ERβ over-expression. Similarly, aberrant methylation on the promoter of the gene encoding for steroidogenic factor 1 (SF1), a transcription factor controlling estrogen production, leads to its over-expression and increases E2 levels in the microenvironment surrounding endometriotic cells [9,14,50,51,52]. Additionally, various animal and plant dietary derivatives such as AA and omega-6 polyunsaturated fatty acid (ω-6) can influence the pro-inflammatory effects of prostaglandin PGE_2_ as well as leukotrienes (LTB4) [47,48]. For example, red meat containing AA and ω6 contributes to the inflammation increase in EMS. Due to high amounts of E2 and estrone (E1), a red meat-rich diet might also boost estrogen levels, and it is therefore not recommended in case of EMS [53].

Furthermore, food of animal origin can also contain some chemical contaminants that act as endocrine disruptors (EDs) leading to hormonal homeostasis unbalance [54,55]. Since PGs are thought to be the primary factors for EMS progression, the suggested diet should work to lower their concentration [28,56]. Among the recommended food there are fish, chia and flaxseed oils, containing the omega 3 (ω-3) compound, eicosapentaenoic acid, and docosahexaenoic acid, which inhibit the conversion of AA to PGE_2_ and LTB4, leading to inflammation inhibition [57]. Other highly recommended foods are those rich in phytochemicals such as carotenoids, flavonoids, and isothiocyanates, with well-known anti-proliferative and anti-inflammatory properties [3,47,57].

### 4.1. Endometriosis Hormonal Imbalance (E2 and P4) and Nuclear Receptors

E2 and P4 regulate the homeostasis and function of the human uterus and its endometrium, ensuring efficient menstrual cycles and fertility [3,10,58]. E2 regulates proliferation of endometrium and supports the growth of endometrial gland before ovulation, preparing the endometrium for P4 activity [9]. P4 inhibits E2 activity, triggering the decidualization process [15]. These hormones act by binding their intracellular nuclear receptors (NRs), the estrogen receptors (ERs) and progesterone receptors (PRs). Two main estrogen receptors (ERs), ERα and ERβ, encoded by two different genes (*ESR1*, *ESR2*) are known [15,59]. On the contrary, the P4 endometrium cells responsiveness is mediated by the coordinated actions of two PRs isoforms, PR-A and PR-B, transcribed from two different promoters of the same gene and sharing a common structure, with only additional 164 amino acid domains at the amino terminus of PR-B [60]. PR expression is induced by E2 trough ERα, and, in turn, PR inhibits ERα expression, creating a feedback system to balance downstream effects [61].

EMS is mainly characterized by high E2 levels and resistance to P4. Loss of P4 responsiveness leads to both increased growth of endometriotic lesions and a non-receptive endometrium, as its signaling is required to counteract E2-induced proliferation and promote decidualization [62]. This imbalance also enhances the recruitment of immune cells promoting inflammation and allowing angiogenesis [11,15,63]. The altered expression of several enzymes involved in E2 biosynthesis contributes to increase the estrogen levels in EMS. Among them, aromatase, which catalyzes the conversion of androgens into estrogens, is over-expressed, while 17β-hydroxysteroid dehydrogenase type 2 (HSD17β2), which is normally induced by P4 and triggers the conversion of E2 in the less potent sex steroid hormone E1, is reduced [63,64]. Furthermore, the steroidogenic acute regulatory protein (StAR), which triggers the ex-novo synthesis of E2 from cholesterol, is upregulated [14,65]. E2 over-production triggers the recruitment of immune cells which further produce inflammatory mediators in endometriosis lesions. This modified microenvironment is in turn able to increase COXs and aromatase expression, further increasing the production of E2 and PGs, leading to severe inflammation. Moreover, the over-expression of COXs and the increased synthesis of PGs is influenced by over-expression of ERβ [59,66]. In a normal endometrium, PR-A and PR-B are expressed both in stromal and epithelial cells, but the loss or down-regulation of either one or both isoforms may cause endometrial lesions [67]. Indeed, in EMS, the PR-B expression is significantly reduced while the PR-A expression is significantly higher [68,69], causing P4 resistance and improper retinoid synthesis [9,70].

In normal cells, P4 pathway increases retinoic acid (RA) synthesis in endometrial stromal cells leading to an increase in HSD17β2 expression in endometrial epithelial cells [70]. Although P4 levels are similar in healthy and EMS-affected women, in the presence of bioavailable P4, the P4 resistance hinders PR activation and transcription of P4 target genes [13,41,70]. Then, the inability of endometriosis epithelial cells to express HSD17B2 may decrease the PR-B level in stromal cells, contributing to excessive estradiol production [71].

Accordingly, endometriotic epithelial cells, which do not express HSD17β2, cannot inactivate estradiol [70,72]. Since endometriosis stromal cells show lower ERα and higher ERβ expression with respect to healthy cells, PR-B is completely absent and it is not able to induce HSD17β2 [73].

The increased ERβ expression is strictly associated with the hypomethylation of its promoter, while the decrease in ERα occurs due to the hypermethylation of its promoter and the direct inhibition by ERβ [11]. The increased E2/ERβ ratio can be associated to enhanced lesion survival and inflammation [3] that, by a positive feedback mechanism, stimulates COX-2 and increases PGE_2_ production. Concluding, estrogens, initializing the PGs production, strongly impact EMS progression, since the inhibition of the PGs biosynthesis decreases the pathology incidence as well as EMS-related inflammatory conditions, chronic pelvic pain and infertility [9].

### 4.2. Inflammatory Pathways in Endometriosis

**Figure 3:**
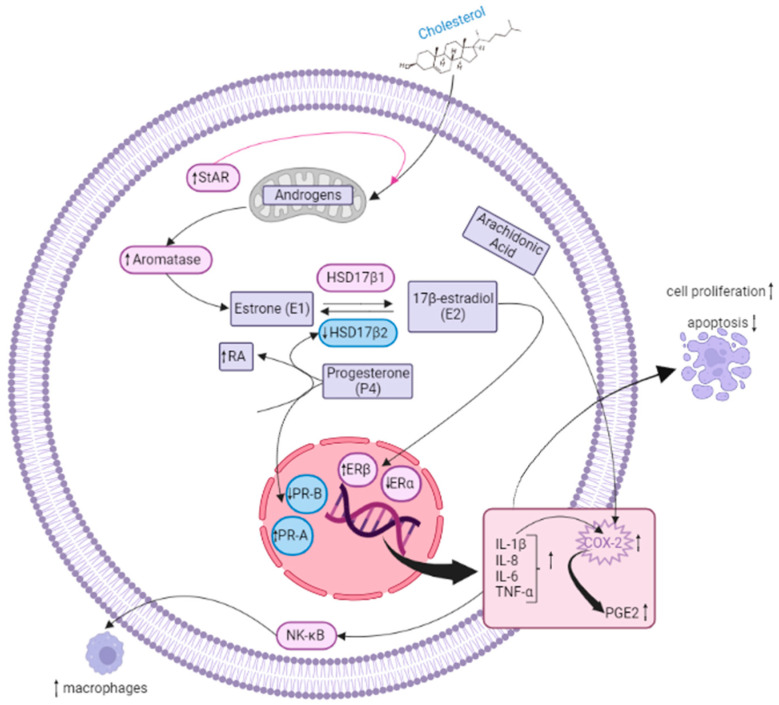
Molecular and inflammatory mechanisms driving EMS. The figure depicts the main alterations occurring in EMS. In particular, increased levels of CYP19A1 and StAR enzymes, together with reduced HSD17β2, boost E2 production. Concomitantly, ERβ over-expression triggers COXs expression/activation and PG increase. Resistance to P4, which normally elevates RA levels, hinders HSD17β2 expression in epithelial cells, thus contributing to enhancing E2 levels. Moreover, the rise in ER/ERβ ratio also promotes inflammation by enhancing cytokine expression (IL-1β, IL-8, IL-6, TNFα) and further stimulating COX-2 activity. This latter process metabolizes AA to produce PGE_2_. Inflammation drives NF-κB pathway activation, thus promoting macrophage recruitment. Created with biorender.com.

EMS is now well recognized as an inflammatory disease [15], and increased inflammatory responses in ectopic endometrial implants are thought to be responsible for EMS pathogenesis. Endometriosis implants are characterized by the activation of pro-inflammatory factors and signaling pathways, as well as by the increased infiltration of immune cells such as macrophages. These elements support the lesion survival by enhancing EMS-related inflammation and, as a consequence, increased levels of MMPs, PGs, chemokines, and cytokines are detected in the peritoneum, endometrium and serum of patients [74,75,76]. Among the cytokines, IL-1β may be responsible for the increased proliferation of endometriosis cells, while it does not affect healthy endometrial cells [77]. Moreover, IL-1β enhances IL-6 and IL-8 production; they act synergistically to boost proliferation and decrease the apoptotic rate of endometriosis cells [75,78]. Inflammation also influences the endometriosis lesion vascularization. For instance, increased levels of IL-1β boost the shedding of intercellular adhesion molecule-1 (ICAM-1) from peritoneal mesothelial cells indicating a role in the neovascularization mediated by IL-6 and vascular endothelial growth factor (VEGF) [79,80]. IL-1β also regulates COX-2 expression; indeed, the ectopic endometriosis cells are more sensitive to the cytokine stimulation in terms of COX-2 expression with respect to normal cells (Figure 3) [22]. As discussed above, COX-2 is highly expressed in EMS, and COX-2-derived PGE_2_ biosynthesis is closely related to EMS. This pathway may be involved in the regulation of ectopic implantation and growth of the endometrium as well as in the angiogenesis and immunosuppression [22,81]. Notably, the COX-2/PGE_2_ are targeted by NSAIDs [15].

The increase in TNFα levels in EMS may stimulate adhesion and proliferation of endometrial cells on ectopic sites. Moreover, TNFα and IL-1β activate the NK-κB signaling pathway [80], which in turn controls the expression of cytokines and chemokines such as IL-1, IL-6, IL-8, TNF-α, as well as ICAM-1 [81,82] able to boost inflammation and COX-2 expression in endometriosis implants. NF-κB signal transduction stimulates macrophage recruitment [15,22,81]. Furthermore, it is interesting to note that macrophages have been abundantly associated with EMS, enhancing the establishment, progression and angiogenesis in endometriosis implants and promoting the release of different cytokines/chemokines and growth factors (e.g., VEGF) [83,84,85]. Lesion resident macrophages derive from eutopic endometrial tissue, but EMS continuously recruits monocyte-derived macrophages [86]. These cells also play a pivotal role in lesion innervation and nerve fiber sensitization, thus contributing to the pain condition [87,88]. Chemokines, like C-C motif chemokine ligand 2 (CCL2) and 5 (CCL5) which play a pivotal role in macrophage recruitment, are significantly increased in endometriosis lesions [89]. Accordingly, Hogg et al. 2021, demonstrated that endometrial macrophage depletion decreases the endometriosis lesion size in mice [86].

## 5. Natural Substance Anti-Inflammatory Properties

NSAIDs, which act by inhibiting the COX-1 and COX-2 activity and, consequently, by decreasing the PGs levels, are commonly used for EMS treatment [22,90]. These drugs are classified in COX-2-selective and -non-selective NSAIDs [91]. However, both drug categories exhibit advantages and disadvantages. Indeed, the use of COX-1 inhibitors has been associated with severe side effects, such as gastrointestinal bleeding and gastric mucosa damage [91], whereas COX-2-selective NSAIDs, which do not show the above side effects, have been associated with cardiovascular toxicity [92]. In light of this, an increasing attention has been recently focused on the use of alternative natural substances such as polyphenols to treat the EMS pain and inflammation [3,93].

### 5.1. Flavonols

**Figure 4:**
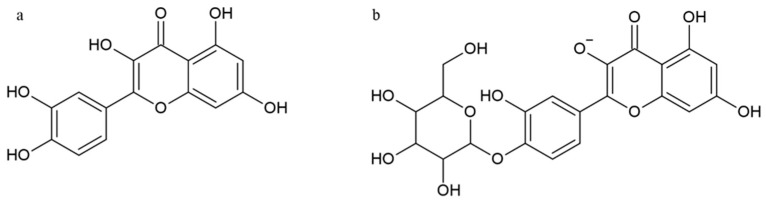
Structure of quercetin (**a**) and quercetin-4′-*O*-β-d-glucoside (**b**).

One of the most abundant flavonols in fruits and vegetables such as onion, cauliflower, apple, berry and chili pepper is quercetin (QRC, 3,3′,4′,5,7-pentahydroxyflavone), well known for its anti-cancer, anti-allergic and anti-inflammatory properties [57,94]. The most common flavonols in plants are glycosides, although aglycones are generally also present (Figure 4).

The phosphoinositide 3-kinase (PI3-K)/Akt/mammalian target of the rapamycin (mTOR) pathway has a pivotal role in tumorigenesis, angiogenesis, tumor growth and metastasis [95,96]. It has been found that QRC inhibits the AKT/mTOR pathway exerting anti-cancer effect by reducing cancer cell viability and enhancing apoptosis and autophagy [97]. Moreover, QRC inhibits cell proliferation and induces cell cycle arrest and apoptosis in endocervical cell lines VK2/E6E7 and End1/E6E7. QRC has anti-inflammatory activity correlated with the inhibition of AA and the production of inflammatory mediators such as PGs and leukotrienes that are also involved in the regulation of uterine contractile activity [98]. Accordingly, Signorile et al., by using a QRC-based dietary supplement to treat EMS patients for three months, found considerably lower serum PGE_2_ levels [99].

### 5.2. Flavones

**Figure 5:**
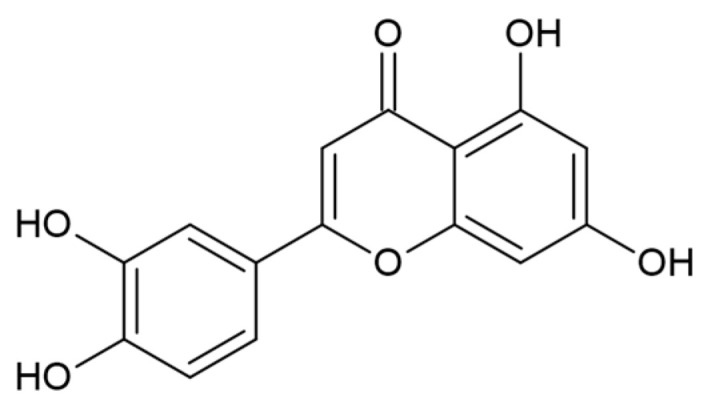
Structure of luteolin.

Luteolin (3′,4′,5,7-tetrahydroxyflavone, LUT) is a natural flavone which can be found in different plants, nuts, and herbs [100,101]. Chemically, it is composed of a C6-C3-C6 carbon skeleton with two benzene rings linked by a heterocyclic ring [102] (Figure 5).

The absence of the hydroxyl group on C3 distinguishes flavones from flavonols [103]. Dietary sources rich in LUT include several vegetables, such as carrot, broccoli, parsley, olive, thyme and clove. The active metabolites or derivatives of LUT, such as luteolin-glucuronide and luteolin 7-glucoside, are known to have anti-oxidant, anti-tumoral, anti-apoptotic, anti-microbial and anti-inflammatory properties [101,104]. The anti-inflammatory activity of LUT is achieved at micromolar concentrations by inhibiting the expression of COX-2 and the production of several pro-inflammatory mediators such as TNF-α and IL-6, hindering macrophage activation and release of excessive amounts of PGs [101,105]. Moreover, LUT can also regulate several signal transduction pathways, including NF-κB, AP1 and JAK–STAT [106], and it can inhibit macrophage recruitment to the endometriotic lesions by suppressing the secretion of CCL2 and CCL5 by endometriosis cells [101]. In a recent molecular docking study, it has been shown that LUT inhibits inflammation in EMS acting on signal transducer and activator of transcription 3 (STAT3), phosphoinositide-3-kinase regulatory subunit 1(PIK3R1), and mitogen-activated protein kinase 1 (MAPK1), also regulating MAPK, PI3K, TNF, and NF-κB signal transduction [107].

### 5.3. Isoflavones

**Figure 6:**
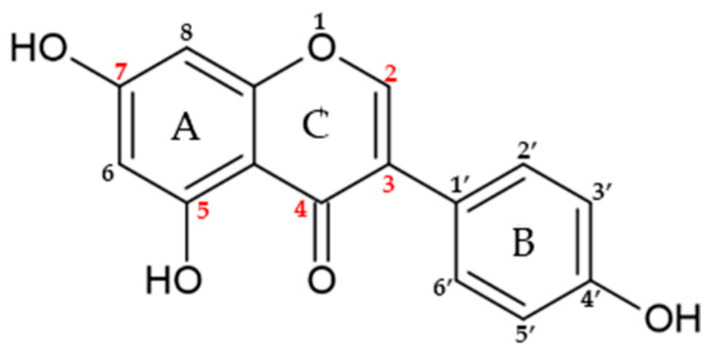
Structure of genistein.

Genistein (5,7-dihydroxy-3-(4-hydroxyphenyl) chromen-4-one, GEN) is an isoflavone found in soybeans, soy-derived foods and other legumes (Figure 6).

Interestingly, GEN has a molecular structure comparable to that of mammalian estrogens and has a 20-fold stronger affinity for ERβ than for ERα [108].

The chemical structure of GEN consists of 15 carbons arranged in two aromatic rings (A and B) and linked by another carbon pyran ring composed of the 3-phenylchromen-4-one nucleus [109]. Moreover, the structure of GEN presents a double bond between positions two and three, and possess an oxo group at position four of ring C, together with three additional hydroxyl groups at positions five and seven of the A ring and position four of the B ring [110]. GEN has pharmacological activities and works as an anti-angiogenic, anti-proliferative, anti-oxidant and anti-inflammatory drug, which can exert several health effects on human health. It is a pleiotropic molecule able to interact with different cellular targets involved in inflammation [111]. The GEN anti-inflammatory properties are exerted through several pathways such as the down-regulation of NF-κB, which promotes the reduction of IL-6, IL-1, TNF-α, TNF-β [35,112,113]. AMP-activated protein kinase (AMPK) is known to inhibit inflammation by decreasing NF-κB levels and pro-inflammatory markers [114]. GEN also reduces inflammation through AMPK activation and subsequent NF-κB suppression [115]. Moreover, GEN inhibits the effect of MAPK pathways [109], which is activated by most of inflammatory stimuli [109]. In addition, the down-regulation of the cytokine-induced signal transduction pathways in the immune system cells can be affected by GEN [116]. A link between GEN and the COX-2/PGE_2_ pathway has also been found. Indeed, GEN can inhibit LPS-induced COX-2 expression and PGE_2_ production in macrophages [22,113]. Therefore, this compound potentially could inhibit EMS development and it can be potentially used as an anti-inflammatory natural treatment for EMS.

### 5.4. Stilbenoids

**Figure 7:**
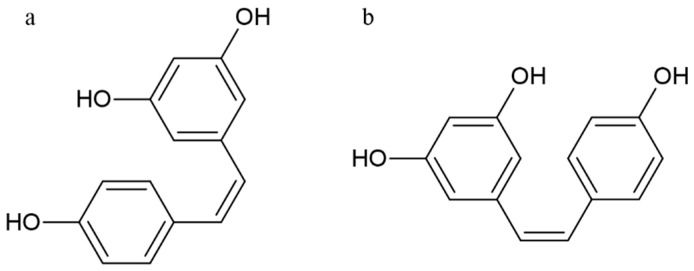
Structure of cis-resveratrol (**a**) and trans-resveratrol (**b**).

RESV(trans-3,4,5-trihydroxystilbene) is a natural polyphenol belonging to the family of stilbenoids, highly concentrated in grape, wine, tea, peanut and berry, and playing an important role in a wide range of biological activities [117]. RESV has anti-tumor and anti-inflammatory properties, as well as exhibits anti-oxidative, anti-microbial, and estrogenic activities [117,118]. The molecule presents two aromatic rings linked to each other by a double ethylene bridge and two aromatic rings linked to each other by a double ethylene bridge. This chemical structure can be therefore present in two isomeric forms, cis-resveratrol and trans-resveratrol, respectively (Figure 7) [119].

RESV anti-inflammatory activity has been well documented in different cancer cell lines, and it is based on different mechanisms by which it inhibits different inflammatory pathways such as COX-2, NF-κB and activator protein 1 (AP1) [117,120]. Particularly, RESV inhibits NF-κB pathway blocking the activation of several pro-inflammatory cytokines such as IL-1β [31]. Moreover, RESV decreases the secretion of pro-inflammatory cytokines (e.g., IL-6, IL-8, and TNF-α) and the expression of adhesion proteins, such as intercellular adhesion molecule (ICAM)-1 [121,122]. RESV is also able to induce, in a concentration-dependent manner, the suppression of IL-1α, IL-6 and TNFα, and the down-regulation of both mRNA expression and IL-17 protein levels [123]. In EMS, AP1 is involved in the transcription of various biomolecules and pro-inflammatory cytokines (e.g., IL-2, IL-3, IL-4, IL5-, IL-13). RESV-dependent AP1 reduction promotes the indirect inhibition of COX-2 activity [31]. Furthermore, RESV interacts with the AA pathway, suppressing the COX-2 effects through the inhibition of PMA-induced COXs transcription in mammary epithelial cells due, in turn, to the protein kinase C pathway inhibition [31,117]. Finally, RESV inhibits COX-2 promoter activity which is mediated by ERK-1 and c-Jun. Therefore, in EMS, the anti-inflammatory activity of RESV is exerted by the PGs synthesis inhibition for a direct COX enzyme synthesis down-regulation activity, as well as by the direct inhibition of activated immune cell sand pro-inflammatory cytokine release [31,117,124].

## 6. Conclusions

The developing pathogenic and physiologic processes that involve the mechanisms of endometriosis are extremely heterogeneous and have not yet been fully elucidated. This implies that the therapeutic approaches of intervention to treat this pathology are limited. Therefore, the use of new therapeutic agents for the treatment of endometriosis turns out to be necessary. Their anti-inflammatory action and their potential phytoestrogenic effect can modulate estrogen networks without causing serious adverse effects unlike conventional anti-estrogenic therapy. Polyphenols may represent new therapeutic agents for the treatment of endometriosis aimed at improving the living conditions of women affected by this disease. The use of polyphenolic compounds for endometriosis treatment have no negative effects on fertility, reproductive organs and development of offspring; moreover, it is more convenient than the use of conventional treatment and turns out to be more suitable for long-term treatment.

## Data Availability

Data sharing not applicable.
